# *BRCA1* haploinsufficiency cell-autonomously activates RANKL expression and generates denosumab-responsive breast cancer-initiating cells

**DOI:** 10.18632/oncotarget.16558

**Published:** 2017-03-25

**Authors:** Elisabet Cuyàs, Bruna Corominas-Faja, Martín Muñoz-San María, Begoña Martin-Castillo, Ruth Lupu, Joan Brunet, Joaquim Bosch-Barrera, Javier A Menendez

**Affiliations:** ^1^ Program Against Cancer Therapeutic Resistance (ProCURE), Metabolism and Cancer Group, Catalan Institute of Oncology, Girona, Catalonia, Spain; ^2^ Molecular Oncology Group, Girona Biomedical Research Institute (IDIBGI), Girona, Catalonia, Spain; ^3^ Neuroimmunology and Multiple Sclerosis Unit, Dr. Josep Trueta University Hospital, Girona Biomedical Research Institute (IDIBGI), Girona, Catalonia, Spain; ^4^ Unit of Clinical Research, Catalan Institute of Oncology, Girona, Catalonia, Spain; ^5^ Mayo Clinic, Department of Laboratory Medicine and Pathology, Division of Experimental Pathology, Rochester, MN, USA; ^6^ Mayo Clinic Cancer Center, Rochester, MN, USA; ^7^ Deparment of Medical Oncology, Catalan Institute of Oncology, Girona, Catalonia, Spain; ^8^ Department of Medical Sciences, Medical School, University of Girona, Girona, Catalonia, Spain

**Keywords:** denosumab, RANK, RANKL, cancer stem cells, BRCA1

## Abstract

Denosumab, a monoclonal antibody to the receptor activator of nuclear factor-κB ligand (RANKL), might be a novel preventative therapy for *BRCA1*-mutation carriers at high risk of developing breast cancer. Beyond its well-recognized bone-targeted activity impeding osteoclastogenesis, denosumab has been proposed to interfere with the cross-talk between RANKL-producing sensor cells and cancer-initiating RANK^+^ responder cells that reside within premalignant tissues of *BRCA1*-mutation carriers. We herein tested the alternative but not mutually exclusive hypothesis that *BRCA1* deficiency might cell-autonomously activate RANKL expression to generate cellular states with cancer stem cell (CSC)-like properties. Using isogenic pairs of normal-like human breast epithelial cells in which the inactivation of a single *BRCA1* allele results in genomic instability, we assessed the impact of *BRCA1* haploinsufficiency on the expression status of RANK and RANKL. RANK expression remained unaltered but RANKL was dramatically up-regulated in *BRCA1^mut/+^* haploinsufficient cells relative to isogenic *BRCA1^+/+^* parental cells. Neutralizing RANKL with denosumab significantly abrogated the ability of *BRCA1* haploinsufficient cells to survive and proliferate as floating microtumors or “mammospheres” under non-adherent/non-differentiating conditions, an accepted surrogate of the relative proportion and survival of CSCs. Intriguingly, CSC-like states driven by epithelial-to-mesenchymal transition or HER2 overexpression traits responded to some extent to denosumab. We propose that breast epithelium-specific mono-allelic inactivation of *BRCA1* might suffice to cell-autonomously generate RANKL-addicted, denosumab-responsive CSC-like states. The convergent addiction to a hyperactive RANKL/RANK axis of CSC-like states from genetically diverse breast cancer subtypes might inaugurate a new era of cancer prevention and treatment based on denosumab as a CSC-targeted agent.

## INTRODUCTION

Two recent studies have provided strong evidence that denosumab, a fully humanized monoclonal antibody that binds and inactivates the receptor activator of nuclear factor-κB ligand (RANKL), might be a novel preventative therapy for carriers of *BRCA1/2* mutations, a group of woman predisposed to high lifetime risks of breast and ovarian cancer [1, 2]. Denosumab, by blocking osteoclast maturation, function, and survival, is currently used for the treatment of postmenopausal osteoporosis, cancer treatment-induced bone loss, and skeletal complications of malignancies [3–6]. If proven to reduce the incidence of *BRCA1/2*-related carcinomas in clinical trials, the repurposing of a bone-targeted agent such as denosumab for oncology indications might inaugurate a new era of molecularly targeted pharmacological cancer prevention therapies and perhaps cancer treatments using RANKL inhibitors for millions of people worldwide [7].

The study by Penninger and colleagues demonstrated that genetic inactivation of RANKL in mammary epithelium was sufficient to significantly delay tumor onset, reduce incidence, and attenuate tumor progression in multiple models of mammary carcinogenesis [1]. Moreover, long-term subcutaneous administration of a RANKL-targeting antibody fragment (RANK-Fc) almost completely prevented the spontaneous development of pre-neoplastic lesions due to *BRCA1* deficiency [1]. The findings by Lindeman and co-workers using luminal progenitor cells from histologically normal tissue obtained in the pre-neoplastic phase from carriers of *BRCA1* mutations revealed that highly proliferative, genomically unstable RANK^+^ cells were the key target cancer-driven population in this high-risk group [2]. Pharmacological inhibition of RANKL in *BRCA1*-deficient mouse models using the RANKL inhibitor OPG-Fc or a RANKL-specific monoclonal antibody significantly delayed tumor onset, confirming the therapeutic value of targeting the RANKL/RANK pathway to prevent breast oncogenesis in carriers of *BRCA1* mutations [2]. Importantly, preliminary findings from a small cohort of patients recruited in the “*BRCA-D*” pilot window study, which aims to evaluate the biological effects of denosumab on normal breast tissue from carriers of *BRCA1* and *BRCA2* mutations and high-risk, non-*BRCA* carriers [8], revealed for the first time that RANKL inhibition by denosumab significantly attenuated breast epithelial cell proliferation in carriers of *BRCA1* mutations.

While the aforementioned landmark studies provide genetic and pharmacological models supporting RANKL-targeted approaches as novel preventative strategies for delaying and possibly eliminating the need for existing risk-reducing approaches in carriers of *BRCA1/2* mutations, such as tamoxifen treatment, prophylactic mastectomy and salpingo-oophorectomy [9, 10], the ultimate mechanisms coupling RANKL blockade with impaired initiation of breast tumorigenesis remained largely unexplored. Based on the well-known relationship between altered progesterone signaling and increased RANKL activity [11–16], it was suggested that denosumab might block mitogenic cross-talk between progesterone “sensor” cells (i.e., mature ductal cells) and the hyperactive RANK^+^ luminal “responder” progenitors residing within premalignant tissues of carriers of *BRCA1* mutations [2]. When the Penninger & Lindeman groups reported their findings, our group was evaluating the alternative but not mutually exclusive hypothesis that RANKL/RANK signaling might operate as a molecular mechanism critical for cell-autonomous maintenance and survival of cellular states with cancer stem cell (CSC)-like properties, including self-renewal, tumor-initiation, drug resistance, and metastasis properties.

To evaluate whether *BRCA1* deficiency might cell-autonomously activate RANKL expression to generate RANKL-addicted CSC-like cellular states, we employed isogenic pairs of nontumorigenic, normal-like human breast epithelial cells in which a knock-in of the *185delAG* mutation in a single *BRCA1* allele results in genomic instability and accurately mimics the cell-autonomous consequences of one-hit *BRCA1* inactivation occurring in the breast epithelium of carriers of *BRCA1* mutations [17–19]. To evaluate whether hyperactive RANKL/RANK signaling might be essential for the generation and maintenance of CSC-like cellular states in *BRCA1* haploinsufficient cells, we took advantage of the functional ability of breast cancer cell lines to display a subpopulation of cells with CSC-like properties defined experimentally by their ability to to self-renew and form anchorage-independent multicellular microtumors or “mammospheres” in non-adherent, non-differentiating conditions *in vitro* at low frequency [20, 21]. The mammosphere platform was employed to assess the potential of denosumab as an anti-CSC agent not only in *BRCA1* haploinsufficient cells but also in genetically diverse breast cancer subtypes in which CSC-like states are known to be driven by molecular traits such as epithelial-to-mesenchymal transition (EMT) or HER2-oncogene overexpression (22–30). We now report the ability of denosumab to efficiently target tumorsphere-initiating, RANKL-addicted CSC-like cells in cancer-prone *BRCA1*^mut/+^ breast epithelial cell populations, and also in the presence of key amplifiers of breast cancer CSC-like cellular states including EMT phenomena and HER2 activation.

## RESULTS

### *BRCA1* haploinsufficiency leads to the specific up-regulation of RANKL but not RANK

To investigate the functional importance of the RANKL/RANK signaling pathway in phenotypically relevant models of early tumorigenesis in *BRCA1^mut/+^* carriers, we used a well-defined experimental system of spontaneously immortal and genetically stable, non-tumorigenic MCF10A breast epithelial cells. A common *BRCA1* pathogenic mutation, *185delAG*, a 2-bp deletion in the coding region close to the N-terminus is introduced into *BRCA1* by gene targeting, leading to haploinsufficiency (*BRCA1^185delAG/+^*) and genomic instability [19].

**Figure 1:**
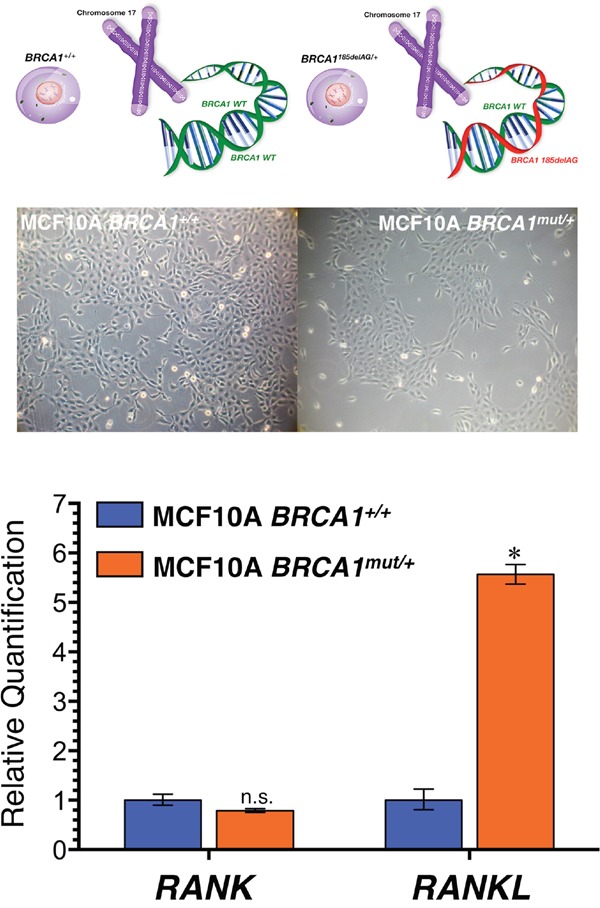
Mutation of a single allele of the cancer susceptibility gene *BRCA1* leads to activation of RANKL expression in normal-like breast epithelial cells *Top*. Inactivating mutation (*185delAG*) of a single *BRCA1* allele leads to haploinsufficiency, which results in genomic instability in the spontaneously immortalized MCF10A cell line. *Bottom*. Total RNA from *BRCA1^+/+^* and *BRCA1^mut/+^* MCF10A isogenic cell pairs was characterized in technical triplicates for the abundance of *RANK (TNFRSF11A*, Hs00921372_m1) and *RANKL* (TNFSF11, Hs00243522_m1) relative to housekeeping genes *GADPH* (Hs99999905_m1) and *18S* (Hs99999901_s1). The transcript abundance was calculated using the delta Ct method and presented as relative quantification (RQ). *p<0.05.

To assess whether *BRCA1* deficiency might suffice to cell-autonomously alter the RANKL/RANK axis in breast epithelial cells, we first assessed the impact of *BRCA1* haploinsufficiency on the expression status of RANK and RANKL molecules in MCF10A cells by RT-PCR. As shown in Figure 1, RANKL expression was dramatically up-regulated (>5-fold) in *BRCA1^mut/+^* cells relative to isogenic *BRCA1^+/+^* parental counterparts. The expression status of RANK, however, remained unaltered in *BRCA1* haploinsufficient cells (Figure 1).

### Denosumab prevents tumorsphere formation in RANKL-overexpressing *BRCA1^mut/+^* human breast epithelial cells

**Figure 2:**
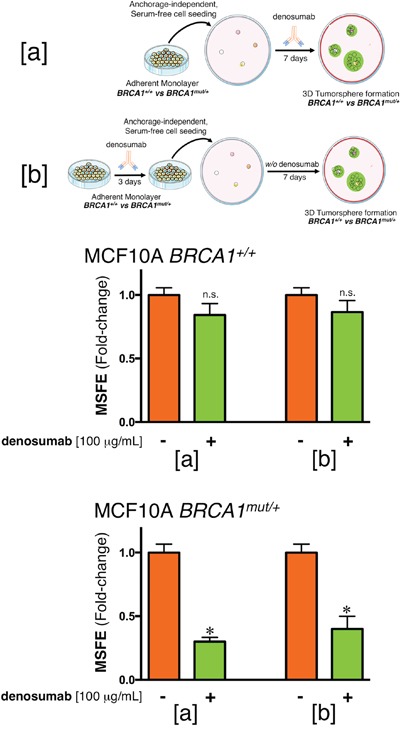
Denosumab significantly reduces mammosphere formation in *BRCA1^mut/+^* breast epithelial cells **(a)** Mammosphere-forming efficiency (MSFE) of *BRCA1^+/+^* and *BRCA1^mut/+^* MCF10A isogenic cell pairs in the absence or presence of 100 μg/mL denosumab was calculated after 7 days and expressed as a percentage (mean ± SD); three technical replicates per n; n = 3 biological replicates. The MSFE of vehicle-alone control cells was normalized to one in each isogenic model. Re-feeding of mammosphere cultures with denosumab and/or sphere medium was performed on day 4. *p<0.05. **(b)** Monolayers of MCF10A *BRCA1^+/+^* and *BRCA1^mut/+^* cells were pre-treated with 100 μg/mL denosumab for 3 days, trypsinized and re-plated for mammosphere assays in the absence of denosumab. The MSFE of vehicle-alone control cells was normalized to one; three technical replicates per n; n = 3 biological replicates. *p<0.05.

Having evidence that *BRCA1^mut/+^* cells become RANKL overexpressors, we next assessed whether a neutralizing anti-RANKL antibody such as denosumab might prevent their tumor-initiating capacity. We first tested the ability of isogenic *BRCA1^+/+^* and *BRCA1^mut/+^* cells to survive and proliferate as floating colonies in serum-free, anchorage-independent conditions, a widely employed *in vitro* assay of the self-renewal and tumor-initiating capacity of CSC-like cells [17, 18]. Cells were treated or not with denosumab and the mammosphere-forming efficiency (MSFE) was calculated as the number of sphere-like structures (diameter >50 μm) divided by the original number of cells seeded. No differences in MSFE were observed for *BRCA1^+/+^* parental cells treated or not with denosumab for 7 days (Figure 2). By contrast, MSFE was significantly lower (70%) in *BRCA1^mut/+^* cells treated with denosumab than in untreated cells. These results suggest that mammosphere-initiating CSC-like cells within *BRCA1^mut/+^* cell populations are differentially more sensitive to the RANKL-targeting effects of denosumab than are those in isogenic *BRCA1^+/+^* cell populations.

When cell monolayers were pre-treated or not with denosumab for 3 days, trypsinized and re-plated for mammosphere assays in the absence of denosumab, no differences were observed in the mammosphere-forming ability of *BRCA1^+/+^* parental cells. Conversely, pre-treatment with denosumab of *BRCA1^mut/+^* cells notably lowered (60%) their MSFE (Figure 2). These results show that the anti-RANKL activity of denosumab appears to preferentially target *BRCA1^mut/+^* cellular states with tumorsphere-initiating capacity even in the presence of a majority of non-CSC like cellular states in the setting of an attached monolayer. Denosumab-mediated suppression of mammosphere-initiating, CSC-like cells was not attributable to a general inhibition of cell proliferation and survival because cell viability measured by MTT reduction in *BRCA1^+/+^* and *BRCA1^mut/+^* cell monolayers was not significantly affected by exposure to denosumab (data not shown).

## DISCUSSION

Based on the recent landmark studies by the Penninger & Lindeman groups [1, 2], clinical trials are warranted to test the efficacy of the anti-RANKL antibody denosumab to prevent breast/ovarian tumorigenesis in carriers of *BRCA1*/*2* mutations, and likely in women with non-*BRCA1/2* mutations at very high risk of developing breast/ovarian cancer. Using a monoallelic *BRCA1* germline mutation model that accurately mimics the molecular events of rapid and tissue-specific predisposition of breast cancer development associated with *BRCA1* haploinsufficiency, we demonstrate the ability of denosumab to robustly impair tumorsphere forming ability, a functional marker that correlates with CSC numbers in cancer cell lines and with progenitor activity in nontransformed mammary epithelial cells of *BRCA1^mut/+^* breast epithelial populations. Given the robust tumorsphere-lowering/CSC-targeting output of denosumab in cancer-prone *BRCA1^mut/+^* breast epithelial cells and the unresponsiveness to denosumab of CSC-driven tumorsphere formation in *BRCA1^+/+^* isogenic cells, and considering also the absence of any confounding effect from different genetic backgrounds in the distinctive and specific anti-CSC activity of denosumab against *BRCA1^mut/+^* cell populations, these findings provide unbiased evidence to suggest that *BRCA1* haploinsufficiency is sufficient to generate CSC-like states that functionally depend on the occurrence of hyperactive autocrine/paracrine RANKL/RANK signaling. Indeed, it is noteworthy that not all the cells within *BRCA1^mut/+^* populations are necessarily addicted to the same extent to autocrine/paracrine RANKL/RANK signaling, which appears to necessarily and exclusively operate in those cell states with tumor-initiating capacity since denosumab treatment does not affect cell viability or proliferation of 2D *BRCA1^mut/+^* cultures but efficiently reduces the mammosphere forming capability of CSC-like cellular states, including those that might pre-exist in 2D monolayers.

The different degrees of local production of RANKL, its specific reception by RANK, and/or the potency of RANKL/RANK signaling might dictate the degree of indispensability *versus* dispensability of the autocrine/paracrine RANKL/RANK pathway in CSC-like *versus* non-CSC cellular states, respectively. In other words, the capacity of denosumab to operate as a *bona fide* CSC-targeting agent might depend on the convergent ability of unrelated CSC drivers (e.g., monoallelic loss of *BRCA1*, EMT, HER2 signaling) to activate and maintain hyperactive RANKL/RANK signaling pathway to which the CSC-like cellular states become addicted. Supporting this notion of denosumab-responsive RANKL/RANK-driven stemness, denosumab might also target *de novo* generation of cancer stemness *via* induction of the EMT program or HER2-oncogene overexpression (Box 1).

Box 1 **Denosumab might target SC cellular states across multiple breast cancer subtypes.**

Having confirmed that *BRCA1* haploinsufficiency cell-autonomously activates RANKL expression and generates denosumab-responsive CSC-like cellular states, we preliminary evaluated whether RANKL/RANK signaling could also favor the maintenance of CSC populations in genetically diverse subtypes of breast carcinoma cells.

***Denosumab and EMT-driven CSC-like cellular states***. By activating NF-κB, the RANKL/RANK pathway has been shown to promote cell migration, invasion, and metastasis *via* the induction of EMT in cancer cells [31–37]. Accordingly, RANKL/RANK signaling might promote dedifferentiation processes that induce EMT and “stemness” in normal breast epithelial and breast cancer cells [38], supporting the notion that RANKL/RANK-driven tumor initiation, progression, and metastasis relies on its ability to regulate self-renewal and activity of CSC-like cells with tumor- and metastasis-initiating capacity [38–41]. To explore whether RANKL might function as a molecular link between EMT and breast cancer stemness, the RANKL-targeting activity of denosumab was studied against the background of the landmark observation that breast cancer cells experimentally induced into EMT dramatically increase the proportion of proliferative CSC-like cells, serving as a valuable screening platform for identifying agents specifically targeting CSCs [22, 23].

First, exploiting the observation that EMT increases the proportion of CSC-like cells (˜100-fold) within breast cancer cell populations, we employed V12H-RAS-transformed derivatives of immortalized mammary epithelial cells driven to undergo EMT by E-cadherin knockdown to assay the ability of denosumab to selectively reduce the EMT-driven enrichment of CSC-like cells [22–26]. We first confirmed previous results showing that the tumorsphere-forming ability of HMLER^shECad^ cells is vastly superior to that of HMLER^shCntrl^ cells, which mostly failed to form *bona fide* mammospheres (data not shown). Of note, the EMT-promoted spheroid formation capacity of HMLER cells was apparently reduced in the presence of denosumab (Figure Box 1A).

**Figure 3:**
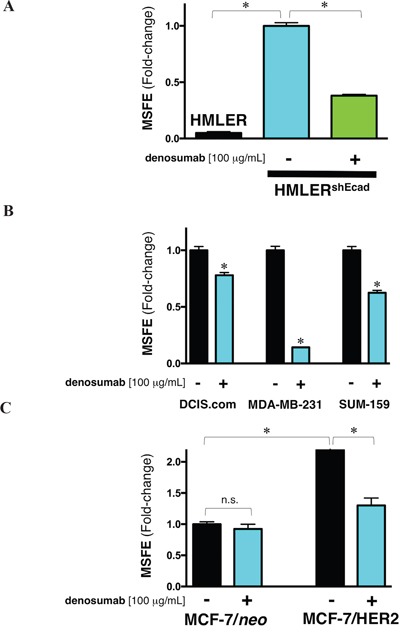
(A) Denosumab reduces the EMT-driven tumorsphere formation ability of *RAS*-transformed human breast epithelial cells. Mammosphere-forming efficiency (MSFE) of HMLER and HMLER^shEcad^ cells in the absence or presence of 100 μg/mL denosumab was calculated after 7 days and expressed as a percentage (mean ± SD); three technical replicates per n; n = 2 biological replicates. The MSFE of vehicle-alone HMLER^shECad^ control cells was normalized to one. Re-feeding of mammospheres cultures with denosumab and/or sphere medium was performed on day 4. *p<0.05. **(B) Denosumab reduces mammosphere formation in basal-like and claudin-low triple negative breast cancer cells**. Mammosphere-forming efficiency (MSFE) of MCF10DCIS.com (*left*), MDA-MB-231 (*middle*), and SUM-159 (*right*) cells in the absence or presence of 100 μg/mL denosumab was calculated after 7 days and expressed as a percentage (mean ± SD); three technical replicates per n; n = 2 biological replicates. The MSFE of vehicle-alone control cells was normalized to one in each model. Re-feeding of mammospheres cultures with denosumab and/or sphere medium was performed on day 4. *p<0.05. **(C) Denosumab reduces HER2-driven augmentation of mammosphere-initiating CSC-like cells in luminal breast cancer cells**. Mammosphere-forming efficiency (MSFE) of the HER2-negative (MCF-7/*neo*) and HER2-overexpressing (MCF-7/HER2) isogenic cell pair in the absence or presence of 100 μg/mL denosumab was calculated after 7 days and expressed as a percentage (mean ± SD); three technical replicates per n; n = 2 biological replicates. The MSFE of vehicle-alone HER2-negative MCF-7 cells was normalized to one. Re-feeding of mammospheres cultures with denosumab and/or sphere medium was performed on day 4. *p<0.05.

Second, to investigate whether denosumab targets CSC-like cells that have been identified within basal-like ductal carcinoma *in situ* (DCIS) [42–45], we used MCF10DCIS.com cells as an *in vitro* model representative of clinical comedo, basal-like DCIS that behaves as a precursor to invasive basal-like, triple-negative breast cancer (TNBC) [27]. It is notable that MCF10DCIS.com cells cultured as spheroids secrete vast amounts of the oncogenic protein osteopontin [46], a CD44 and integrin ligand known to up-regulate the expression of RANKL [47–49]. Although its effect was less conspicuous than in *BRCA1^mut/+^* and EMT-like HMLER^shECad^ cell models, denosumab slightly but significantly reduced by the capacity for MCF10DCIS.com cells to form mammospheres (Figure Box 1B).

Third, to evaluate the generality of the above-mentioned findings, we assessed whether claudin-low breast subtypes displaying increased activation of the EMT program might also contain CSC-like cells responsive to denosumab. Mammosphere formation ability of MDA-MB-231 and SUM-159 cells, two highly aggressive models for claudin-low TNBC breast cancer [29, 30], was apparently decreased by denosumab relative to their respective untreated controls, and this effect was particularly striking for MDA-MB-231 cells (Figure Box 1B).

***Denosumab and HER2-driven CSC-like cellular states***. We examined whether denosumab treatment might prevent the well-recognized ability of HER2 signaling, even in the absence of *HER2* gene amplification, to expand CSC-like breast cancer populations [50–57]. We previously demonstrated that HER2-negative MCF-7 luminal breast cancer cells engineered to express higher amounts of the HER2 oncoprotein (MCF-7/HER2) present a significantly increased ability over MCF-7 parental cells to form mammospheres [58]. In our hands, denosumab treatment was apparently sufficient to return the exacerbated HER2-driven tumorsphere-initiating capacity to almost baseline levels found in HER2-negative MCF-7/*neo* isogenic parental cells (Figure Box 1C).

The apparent inhibitory effects of denosumab on the acquired mammosphere-forming capacity of stably induced EMT-CSCs (Box 2) might suggest that hyperactive RANKL/RANK signaling not only suffices to induce EMT-like phenomena [31–37], but also that the acquisition and maintenance of CSC-like cellular states might be accompanied by activation of the RANKL/RANK signaling pathway, thus generating a positive feedback loop between the RANKL/RANK axis and EMT-related acquisition of stemness traits. Accordingly, denosumab treatment negatively influenced the number of mammospheres formed by MDA-MB-231 and SUM-159 breast cancer cell lines, two *in vitro* models characterized by an enrichment in EMT and CSC-like characteristics that significantly associate with disease recurrence [30, 59–61]. Denosumab-induced RANKL inhibition might not only prevent breast tumorigenesis in cancer-prone epithelial tissues but also reduce the risk of recurrence and metastasis in aggressive subtypes of breast carcinomas [39]. Indeed, the ability of denosumab to partially reduce the numerical expansion of CSC-like cells occurring in response to high signaling levels of *HER2* [50] might be part of the mechanism that induces the CSC phenotype in non-CSC cells, raising the intriguing possibility of combining anti-RANKL denosumab with anti-HER2 therapeutics such as trastuzumab to prevent the increase of metastasis-initiating CSCs [56, 57]. Yet, because serum denosumab concentrations have been found to range between 17.7 and 20.1 μg/mL at month 6 during subcutaneous administration of 120 mg every 4 weeks to patients with breast cancer and bone metastasis [62], it should be acknowledged that the denosumab concentration employed in our tumorsphere assays exceeded by up to 5-fold the circulating systemic concentrations of denosumab in patient populations.

Box 2 **Bone-targeted agents against breast CSCs: The case of zoledronic acid.**

The therapeutic potential of bone-targeted treatments with bisphosphonates (e.g., zoledronic acid) and denosumab in cancer patients might go beyond prevention of skeletal complications [68–71]. In early-stage breast carcinomas, treatment with zoledronic acid leads to improvements in disease-free and overall survival. Denosumab is associated with improved overall survival in patients with metastatic lung cancer [71, 72]. Whereas the recent studies by Penninger & Lindeman [5, 6] have provided robust evidence for the direct effect of denosumab against cancer cells, it should be acknowledged that earlier preclinical data demonstrated the direct antitumor activity of zoledronic acid. However, although treatment with zoledronic acid has been found to prevent and eliminate mammosphere formation in claudin-low breast cancer cells [73], the ultimate mechanism/s underlying its ability to directly target cancer cells, including CSC-like states, appear to be multi-faceted [74–85]. Because inhibition of RANKL/RANK-driven signaling is one of the mechanisms through which zoledronic acid exerts its effects, we tested the potential anti-CSC effect of zoledronic acid on the self-renewal capacity of EMT-driven CSC-like populations by re-evaluating the ability of HMLER^shECad^ cells to survive and proliferate as floating spherical colonies under non-adherent/non-differentiating conditions in the presence of graded concentrations of zoledronic acid (Figure Box2). The EMT-induced spheroid formation capability of breast epithelial cells was dose-dependently prevented (up to 98% reduction) by zoledronic acid. Importantly, full suppression of mammosphere formation was not due to non-specific toxicity of zoledronic acid because cell viability determined by MTT reduction performed in monolayer cultures remained as high as 80% in the presence of an identical concentration of zoledronic acid (i.e., 10 μmol/L). Moreover, zoledronic acid dose-dependently decreased tumor sphere formation in MDA-MB-231 and SUM-159 cells by 70% and 85%, respectively (Figure Box2). Once again, these decreases in mammosphere-initiating capacity of claudin-low breast cancer cells was not due to non-specific zoledronic acid toxicity because identical concentrations of zoledronic acid had no significant impact on cell viability under adherent culture conditions (data not shown).

**Figure 4:**
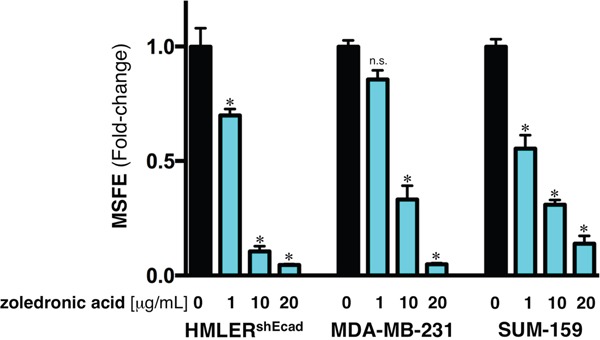
Zoledronic acid reduces mammosphere formation in EMT-enriched breast cancer cell models. Mammosphere-forming efficiency (MSFE) of HMLER^shECad^, MDA-MB-231, and SUM-159 cells in the absence or presence of graded concentrations of zoledronic acid was calculated after 7 days and expressed as a percentage (mean ± SD); three technical replicates per n; n = 3 biological replicates. MSFE of vehicle-alone control cells was normalized to one in each model. Re-feeding of mammospheres cultures with zoledronic acid and/or sphere medium was performed on day 4. *p<0.05.

The propensity for tumor formation in *BRCA1*-associated breast carcinomas appears to be driven by specific molecular and cellular alterations triggered by inherited mutations in *BRCA1* in breast epithelial differentiation before development of cancer. Here, we established that *BRCA1* haploinsufficiency cell-autonomously elicits a vicious cycle involving RANKL/RANK signaling favoring the pool of breast epithelial cells with CSC-like properties [63]. By binding to RANKL, denosumab inhibits the activation of RANK, its only receptor, thus preventing RANKL/RANK interaction and disrupting signaling leading to expansion and maintenance of the CSC population across multiple breast cancer subtypes (Figure 3). Beyond the anticipated primary prevention impact that denosumab-targeted aberrancies in the RANKL pathway might play in *BRCA1* mutation carriers [64], preliminary evidence from the ABCSG 18 trial showing improved disease-free survival of women receiving adjuvant denosumab [65, 66] and currently ongoing clinical investigations of denosumab as an adjunct to neoadjuvant chemotherapy [67] should begin to clarify the role of RANKL blockade to prevent CSC-driven tumor recurrence and metastasis in non-*BRCA1* patients. The observations by the Penninger & Lindeman groups [1, 2] together with our findings suggesting that cell-autonomous activation of the RANKL/RANK signaling axis is a convergently shared, non-oncogenic addiction underlying the generation and maintenance of CSC-like states in response to diverse molecular events such as *BRCA1* haploinsufficiency, EMT phenomena and HER2 activation, might inaugurate a new era of cancer prevention and treatment based on the previously unrecognized CSC-targeted capabilities of bone-targeted agents such as the bisphosphonate zoledronic acid (Box 2) and the anti-RANKL monoclonal antibody denosumab.

**Figure 4:**
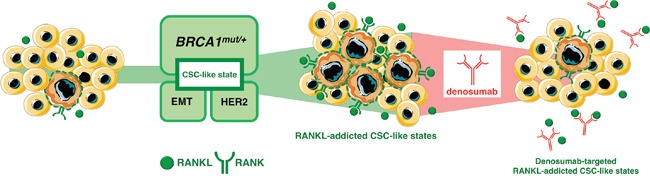
Autocrine/paracrine RANKL/RANK signaling: A shared denosumab-responsive CSC trait across multiple breast cancer subtypes Tumor-initiating cell from genetically diverse pre-cancerous and breast cancer subtypes might share, to a greater (*BRCA1* haploinsufficiency) or lesser extent (EMT) and HER2), a common addiction to hyperactive RANKL/RANK signaling, which allows their survival and self-renewal in a CSC state. This observation might inaugurate a new era of cancer prevention and treatment capable of producing sustained effects based on RANKL-targeting approaches.

### Study limitations

We should acknowledge that our mammospheres studies have used too few cell lines to reliably interpret data in the context of breast cancer-initiating cells derived from molecularly heterogeneous primary human tumors. Additional *ex vivo* experiments using freshly isolated CSC-enriched cell populations from specific *BRCA1* KO animals and primary breast tumor samples of distinct molecular subtypes should definitely clarify the role of the anti-RANKL antibody denosumab as a potential breast CSC-specific inhibitor.

## MATERIALS AND METHODS

### Materials

Denosumab (Prolia^®^) and zoledronic acid (Zometa^®^) were kindly provided by the Hospital Universitari de Girona Dr. Josep Trueta Pharmacy (Girona, Spain).

### Cell lines

Human *BRCA1* (185delAG/+) MCF10A cells with a heterozygous knock-in of a 2-bp deletion of *BRCA1* resulting in a premature termination codon at position 39 and MCF10A isogenic parental cells were obtained from Horizon Discovery Ltd., Cambridge, UK (Cat# HD 101–018 and HD PAR-058, respectively). Cells were routinely grown in DMEM/F-12 (Gibco, Life Technologies, Paisley, UK) including 2.5 mmol/L L-glutamine, and 15 mmol/L HEPES, supplemented with 5% (*v/v*) horse serum (HS), 10 μg/mL insulin, 20 ng/mL hEGF, 0.5 μg/mL hydrocortisone and 0.1 μg/ mL cholera toxin.

HMLER cells expressing a control shRNA (shCntrl) or an shRNA targeting E-cadherin (shEcad) were generated as described [22, 23] and maintained in a 1:1 mixture of DMEM + 10% (*v/v*) heat-inactivated fetal bovine serum (FBS), insulin, hydrocortisone, and Clonetics™ MEGM™ (Mammary Epithelial Cell Growth Medium).

MCF10DCIS.com and SUM-159PT cells were purchased from Asterand (Detroit, MI).DCIS.com cells were cultured in DMEM/F12 with L-glutamine supplemented with 5% HS and penicillin/streptomycin. SUM159PT cells were cultured in Ham's F12 with 5% FBS, 5 μg/mL insulin, and 1 μg/mL hydrocortisone.

MDA-MB-231 cells were purchased from American Type Culture Collection (Manassas, VA) and grown in Improved MEM supplemented with 5% FBS and 2 mmol/L L-glutamine.

HER2-overexpressing MCF-7/HER2 (clone 18) cells and their matched isogenic control (empty vector-transfected) MCF-7/*neo* cells were kindly provided by Mien-Chie Hung (The University of Texas MD Anderson Cancer Center, Houston, TX). Breast cancer cell lines were routinely grown in improved MEM (IMEM; Biosource International, Camarillo, CA, USA) containing 5% (*v/v*) FBS and 2 mmol/L L-glutamine.

All cells were maintained at 37°C in a humidified atmosphere of 95% air/5% CO_2_. Cells were screened periodically for *Mycoplasma* contamination.

### Quantitative real-time polymerase chain reaction (qRT-PCR)

Total RNA was extracted from cell cultures using the Qiagen RNeasy Kit and QIAshredder columns according to the manufacturer's instructions. One microgram of total RNA was reverse-transcribed to cDNA using the Reaction Ready™ First Strand cDNA Synthesis Kit (SABiosciences, Frederick, MD). PCR arrays were processed according to the SABiosciences RT-PCR manual and analyzed using an Applied Biosystems 7500 Fast Real-Time PCR System with an automated baseline and threshold cycle detection. The data were interpreted using the web-based PCR array analysis tool from SABiosciences.

### Mammosphere culture and mammosphere-forming efficiency

Single cell suspensions of cell lines were plated in 6-well tissue culture plates previously coated with poly-2-hydroxyethyl-methacrylate (Sigma, St. Louis, MO) to prevent cell attachment, at a density of 1000 cells/mL in serum-free DMEM/F-12 supplemented with 1% L-glutamine, 1% penicillin/streptomycin, 2% B27 (Invitrogen, Carlsbad, CA), 20 ng/mL EGF (Sigma) and 20 ng/mL FGFb (Invitrogen). The medium was made semi-solid by the addition of 0.5% methylcellulose (R&D Systems, Minneapolis, MN) to prevent cell aggregation. Mammosphere-forming efficiency (MSFE) was calculated as the number of sphere-like structures (diameter >50 μm) formed after 7 days in the absence or presence of denosumab or zoledronic acid, divided by the original number of cells seeded and expressed as a percentage (mean ± SD).

### Cell viability assays

Cell viability was determined using the standard colorimetric MTT reduction assay. For each treatment, cell viability was evaluated as a percentage using the following equation: (OD_570_ of the treated sample/OD_570_ of the untreated sample)×100.

### Statistical analysis

All observations were confirmed by at least three independent experiments. Data are presented as mean ± SD. Two-group comparisons were performed using Student's *t* test for paired and unpaired values. Comparisons of means of ≥3 groups were performed by ANOVA, and the existence of individual differences, in case of significant *F* values at ANOVA, tested by Scheffé's multiple contrasts. *P* values <0.05 were considered statistically significant (denoted as*). All statistical tests were two-sided.
